# National racial/ethnic and geographic disparities in experiences with health care among adult Medicaid beneficiaries

**DOI:** 10.1111/1475-6773.13106

**Published:** 2019-01-09

**Authors:** Steven C. Martino, Megan Mathews, Denis Agniel, Nate Orr, Shondelle Wilson‐Frederick, Judy H. Ng, A. Elizabeth Ormson, Marc N. Elliott

**Affiliations:** ^1^ RAND Corporation Pittsburgh Pennsylvania; ^2^ RAND Corporation Santa Monica California; ^3^ Office of Minority Health Centers for Medicare & Medicaid Services Baltimore Maryland; ^4^ National Committee for Quality Assurance Washington District of Columbia; ^5^ NORC Atlanta Georgia

**Keywords:** CAHPS, disparities, Medicaid, patient experience, race/ethnicity, urbanicity/rurality

## Abstract

**Objectives:**

To investigate whether health care experiences of adult Medicaid beneficiaries differ by race/ethnicity and rural/urban status.

**Data Sources:**

A total of 270 243 respondents to the 2014‐2015 Nationwide Adult Medicaid Consumer Assessment of Healthcare Providers and Systems Survey.

**Study Design:**

Linear regression was used to estimate case mix adjusted differences in patient experience between racial/ethnic minority and non‐Hispanic white Medicaid beneficiaries, and between beneficiaries residing in small urban areas, small towns, and rural areas vs large urban areas. Dependent measures included getting needed care, getting care quickly, doctor communication, and customer service.

**Principal Findings:**

Compared with white beneficiaries, American Indian/Alaska Native (AIAN) and Asian/Pacific Islander (API) beneficiaries reported worse experiences, while black beneficiaries reported better experiences. Deficits for AIAN beneficiaries were 6‐8 points on a 0‐100 scale; deficits for API beneficiaries were 13‐22 points (*P*'s < 0.001); advantages for black beneficiaries were 3‐5 points (*P*'s < 0.001). Hispanic white differences were mixed. Beneficiaries in small urban areas, small towns, and isolated rural areas reported significantly better experiences (2‐3 points) than beneficiaries in large urban areas (*P*'s < 0.05), particularly regarding access to care. Racial/ethnic differences typically did not vary by geography.

**Conclusions:**

Improving experiences for racial/ethnic minorities and individuals living in large urban areas should be high priorities for policy makers exploring approaches to improve the value and delivery of care to Medicaid beneficiaries.

## 1 INTRODUCTION

Medicaid is the primary source of health care for Americans with low income and limited resources, accounting for 17% of total personal health care spending in the United States in 2016.^1^ In 2016, over 70 million people were enrolled in Medicaid, 60% of whom were adults.^2^ Prior to the Affordable Care Act (ACA), low‐income adults were largely excluded from Medicaid, as the median state income eligibility cutoff was 61% of the federal poverty level (FPL) and nonelderly adults were categorically ineligible in most states. The ACA fundamentally changed Medicaid by establishing eligibility for nonelderly adults and enacting a uniform, national income eligibility threshold for this group, 138% of the FPL,^3^ thereby increasing Medicaid enrollment by ~11 million people.^4^ State‐level funding to meet the needs of the Medicaid population has not kept pace with increasing enrollment rates,^5^ prompting policy makers to explore approaches to improve the value and delivery of care to Medicaid beneficiaries.^6^ A key component of providing high‐quality care involves the identification and elimination of disparities based on sociodemographic characteristics.^7^, ^8^ The current study takes a step in that direction by using a newly available, nationally representative dataset to investigate racial/ethnic and urban/rural disparities in care delivered to adult Medicaid beneficiaries.

Numerous investigations of the adult Medicaid population have illuminated racial/ethnic disparities in access to care, utilization of services, receipt of preventive care, and administration of appropriate treatment for a variety of conditions.^9^, ^10^, ^11^, ^12^, ^13^, ^14^ With a few important exceptions,^15^, ^16^ these investigations have not focused on beneficiaries’ experiences with care, in part because of limited data on this important aspect of health care quality. In response to that shortfall, the Centers for Medicare & Medicaid Services (CMS) contracted with NORC at the University of Chicago to conduct the 2014‐2015 Nationwide Adult Medicaid (NAM) Consumer Assessment of Healthcare Providers and Systems (CAHPS) survey. This survey obtained nationwide data on the experiences of care of a large sample of adult Medicaid beneficiaries. Not only is this sample racially and ethnically diverse, it also includes large numbers of beneficiaries living in small towns and rural areas, as well as large and small metropolitan locales.

A variety of factors tied to geography may contribute to health care, including the health care delivery environment, transportation infrastructure, and the distribution of health care resources.^17^, ^18^, ^19^ Although little is known about geographic disparities in care faced by Medicaid beneficiaries, studies of the general U.S. adult population have identified significant barriers to accessing care for rural residents, including provider shortages, recent hospital closures, and long travel distances to see providers.^20^, ^21^, ^22^

In the primarily low‐income Medicaid population, geographic barriers to care may exist in large urban areas as well as rural areas. Recent research has demonstrated substantial growth in high‐poverty urban neighborhoods that are disproportionately occupied by communities of color.^23^, ^24^, ^25^, ^26^ In much the same way that geographic isolation can limit rural residents’ access to high‐quality care,^20^, ^27^ economic and racial segregation can lead to “health care deserts” in urban areas that effectively cut poor people off from high‐quality care that is nearby, but still inaccessible.^28^, ^29^, ^30^ Given multiple disincentives for primary care physicians to live and work in economically depressed urban areas^31^ and some residents’ inability or disinclination to seek care outside of their own neighborhoods, the urban poor may end up disproportionality receiving care from poorer‐performing providers.

There is evidence that geographic factors may exacerbate racial/ethnic disparities in health care.^30^, ^32^, ^33^, ^34^, ^35^ For example, studies of the general adult population have found that racial/ethnic disparities in health care access are generally greater in rural than in urban settings.^33^ Within the Medicaid population, similar synergistic effects of race/ethnicity and geography may be observed among the urban poor. For example, combined racial and economic segregation in large metropolitan areas may make it especially difficult for poor, racial/ethnic minorities to access high‐quality care in these areas.^26^

The current study used data from the NAM CAHPS survey to investigate differences in adult Medicaid beneficiaries’ experiences of care based on (a) race/ethnicity, (b) rural/urban residency, and (c) the combination of these two sociodemographic characteristics. We expected to find racial/ethnic differences that are comparable to ones found in other populations,^36^, ^37^, ^38^ that is, that white beneficiaries would, for the most part, report more favorable experiences with care than racial/ethnic minorities. For the reasons outlined above, we also expected to find a disadvantage for beneficiaries living in rural and large urban areas compared with those living in intermediate areas, and we expected a synergistic effect of race/ethnicity and rural/urban residence on beneficiaries’ experiences with care.

## 2 METHODS

### 2.1 Data source and study population

Data came from the 2014‐2015 NAM CAHPS survey, a nationally representative stratified random sample of adult Medicaid beneficiaries. To be eligible for the survey beneficiaries needed to be age 18 or older on December 31, 2013, and enrolled in Medicaid during October 2013‐December 2013. The sample design excluded institutionalized beneficiaries, those for whom Medicaid pays some of the expenses they incur under Medicare (partial duals), those who qualified for Medicaid via a family planning waiver, those with unknown managed care plans, and those who simultaneously reside in two states or with unknown contact information. The sample design stratified beneficiaries into four mutually exclusive enrollment groups based on program eligibility: adults dually eligible for Medicaid and Medicare (dual‐eligible), non‐dual‐eligible adults with disabilities, non‐dual‐eligible, nondisabled adults enrolled in a managed care organization, and non‐dual‐eligible, nondisabled adults who obtained care from a fee‐for‐service provider or who were enrolled in a primary care case management plan. The survey was administered via mail, with telephone follow‐up of nonrespondents, in English and Spanish. Four waves of data collection occurred from December 2014 through July 2015. A total of 1 205 757 eligible Medicaid beneficiaries were sampled nationwide from 46 states and the District of Columbia (roughly 29 000 per state/district; Alaska, New Hampshire, North Dakota, and Wisconsin did not participate), with 272 679 responding (a 23% overall response rate).

### 2.2 Dependent variables

The dependent variables were four multi‐item composites constructed from 10 NAM CAHPS report items. These composites measured beneficiaries’ own experiences of care in the prior 6 months in the following areas: Getting Needed Care (two items; Cronbach's alpha [α] = 0.64), Getting Care Quickly (two items; α = 0.68), How Well Doctors Communicate (four items; α = 0.90), and Health Plan Information and Customer Service (two items; α = 0.77). Items comprising the composites (see Appendix Table S1 for detail^1^ ) were asked of the subset of beneficiaries to whom they were applicable (screener items were used to assess applicability). All items had the following response options: *never, sometimes, usually,* and *always*. Top‐box scoring was used for the four composite measures for ease of interpretation. In this scoring approach, the most favorable response option (i.e, *always*) is coded 100 and all other options (i.e, *never*,* sometimes*, and *usually*) are coded 0 prior to averaging nonmissing items to create composite scores. For example, the score for a respondent who answered “always,” “always,” “never,” and “sometimes” to the four doctor communication items would be (100 + 100 +0 + 0 = 200)/4 = 50. Scores on the composite measures can be approximately interpreted as the percentage of respondents who gave the most favorable response possible, on average, on the survey scale. Previous analyses of CAHPS scores have suggested that statistically significant differences of 1 point on a 0‐100 scale can be considered small; differences of three points can be considered medium; and differences of five points can be considered large.^39^ For instance, a three‐point increase in some CAHPS measures has been associated with a 30% reduction in disenrollment from health plans, which suggests that even “medium” differences in CAHPS scores may indicate substantially different care experiences.^40^ In describing results below, we will refer to nonsignificant differences on the dependent measures as “similar” scores.

### 2.3 Main predictor variables

#### 2.3.1 Race/Ethnicity

Information on race/ethnicity was primarily collected via self‐report on the NAM CAHPS survey, although in some instances state Medicaid personnel assisted in the response or included administrative, rather than self‐reported values. The cases that were not fully self‐reported cannot be identified. In the survey, respondents were asked to indicate whether they were of Hispanic, Latino, or Spanish origin. Those who responded affirmatively were asked to indicate whether they were Mexican, Puerto Rican, Cuban, or of other Hispanic, Latino, or Spanish origin. Race was measured on the survey using an item with fifteen response options: white; black or African American; American Indian or Alaska Native (AIAN); Asian Indian; Chinese; Filipino; Japanese; Korean; Vietnamese; other Asian; Native Hawaiian; Guamanian or Chamorro; Samoan; other Pacific Islander; or some other race. Our primary set of analyses focused on five mutually exclusive racial/ethnic groups: Hispanic (any beneficiary of Hispanic, Latino, or Spanish origin regardless of race), non‐Hispanic white, non‐Hispanic black, non‐Hispanic AIAN, and non‐Hispanic Asian or Pacific Islander (API; a combination of all Asian and Pacific Islander categories). We refer to beneficiaries in these five categories as Hispanic, white, black, AIAN, and API. Although they are included in our analysis, we do not report effects for multiracial beneficiaries (n* *=* *19 842) because they are a heterogeneous and therefore hard‐to‐interpret group. Focusing our primary analyses on these five categories gave us the statistical power needed to investigate how racial/ethnic differences in experiences with care vary by categories of rurality/urbanicity. To understand variation within these larger racial/ethnic categories, we additionally examined experiences of care by Hispanic and API subgroups. For these analyses, we distinguished four Hispanic subgroups (Mexican, Puerto Rican, Cuban, and other Hispanic) and two API subgroups (Asian and Native Hawaiian or other Pacific Islander [NHOPI]).

#### 2.3.2 Rural/urban status

NORC grouped U.S. counties into four categories using the Rural‐Urban Commuting Area codes developed by the U.S. Department of Agriculture in 2010: large urban areas (i.e, areas containing at least one urbanized area of 50 000 or more people; codes 1‐3), small urban areas (i.e, areas containing at least one urban cluster of <50 000 but at least 10 000 people; codes 4‐6), small towns (i.e, areas containing a small urban cluster of 2500 to 9999 people; codes 7‐9), and rural areas (i.e, areas of 2500 or fewer people that are not adjacent to an urbanized area or urban cluster.^41^

### 2.4 Control variables

Because other beneficiary characteristics are also known to be associated with response tendencies and may differ by race/ethnicity or rural/urban status, we also included age (18‐24, 25‐34, 35‐44, 45‐54 [reference category], 55‐64, 65‐74, and 75 or older), education (eighth grade or less, some high school, high school graduate or General Equivalency Diploma [reference category], some college or 2‐year degree, 4‐year college graduate, and more than a 4‐year college degree), and self‐rated health status (poor, fair, good [reference category], very good, and excellent) in all models.^1^ To control for variation in the quality of care delivered to Medicaid beneficiaries living in different states, 46 state/district indicators were included in the models. Even with these extensive controls, caution is needed when interpreting Asian white differences on CAHPS measures, as there are known differences in response tendency between these groups.^42^

### 2.5 Missing data and imputation

Less than 1% of respondents were missing information on race/ethnicity (n* *=* *2302), urban/rural status (n* *=* *137), or both (n* *=* *2436). Information from these respondents was excluded from the analysis. Missing values on control variables (2%‐3%) were imputed using within‐state and enrollment‐sampling‐stratum means for all nonmissing respondents. Respondents with missing outcome variables were excluded from analyses of those outcomes.

### 2.6 Statistical analysis

The SURVEYREG procedure within the *SAS* 9.4 software package (SAS Institute Inc., Cary, NC, USA) was used to apply weights that accounted for the complex survey design and adjusted for post‐sampling‐ineligible adults and nonresponse at the level of the enrollment stratum within states. We used linear regression with weighted least‐squares estimation to model the association between race/ethnicity and Medicaid beneficiaries’ experiences with care. Each of our primary models (one for each of the four dependent variables) of the association between race/ethnicity and patient experience included four indicators of race/ethnicity (white was the reference group) and the previously described control variables. A second set of models focused on API and Hispanic subgroups. These models were identical to the primary models except that they included two indicators of API subgroup membership (Asian and NHOPI) in place of the single API indicator, and four indicators of Hispanic subgroup membership (Mexican, Puerto Rican, Cuban, and other) in place of the single indicator of Hispanic ethnicity. We modeled the association between rural/urban status and experiences with care using a similar set of analyses. These models included three indicators of rural/urban status (large urban was the reference group) and the previously described covariates as predictors. A final series of models predicted each outcome variable from race/ethnicity (Hispanic, black, AIAN, and API vs white), rural/urban status, the interaction of race/ethnicity and rural/urban status, and the covariates. We conducted joint tests of the interaction coefficients from these final models to assess whether differences in care between white and racial/ethnic minority beneficiaries varied significantly by rural/urban strata.

## 3 RESULTS

Excluding those of unknown race/ethnicity, 53% of beneficiaries were white, 19% were black, 13% were Hispanic, 7% were multiracial, 5% were API, and 2% were AIAN. Table 1 shows how beneficiaries were distributed across the four rural/urban categories. Approximately 70% of beneficiaries resided in large urban areas, 14% in small urban areas, 8% in small towns, and 7% in rural areas; rural residence differed strongly by race/ethnicity. More than a third of AIAN beneficiaries lived in rural areas, compared with approximately 1% of API beneficiaries, 2% of black beneficiaries, 4% of Hispanic beneficiaries, and 10% of white beneficiaries.

**Table 1:** Distribution of 2014‐2015 NAM CAHPS survey respondents^a^ across rural/urban areas, overall and by racial/ethnic group

Rural/urban category^b^	Total states	Total respondents		AIAN		API		Black		Hispanic		White	
	N	N	%	N	%	N	%	N	%	N	%	N	%
Large urban	47	188 784	69.9	2584	40.2	11 521	90.4	43 014	82.4	29 161	82.6	88 483	61.6
Small urban	45	38 914	14.4	819	12.7	856	6.7	5243	10.0	3604	10.2	25 497	17.7
Small town	43	22 682	8.4	793	12.3	189	1.5	2887	5.5	1309	3.7	15 886	11.1
Rural	44	19 863	7.4	2232	34.7	174	1.4	1053	2.0	1248	3.5	13 848	9.6

Notes:

A total of 272 679 Medicaid beneficiaries from 47 states responded to the 2014‐2015 NAM CAHPS survey. A total of 2436 respondents were missing information on race/ethnicity and/or rural/urban status and thus were excluded from this analysis. Multiracial beneficiaries are included in the “total respondents” category, but are not shown as a separate racial/ethnic group. Geographic groups are based on Rural‐Urban Commuting Area codes: large urban (codes 1‐3), small urban (codes 4‐6), small towns (codes 7‐9), and rural (code 10).

AIAN, American Indian or Alaska Native; API, Asian or Pacific Islander; NAM CAHPS, Nationwide Adult Medicaid Consumer Assessment of Providers and Systems.

a N's are unweighted counts of respondents; percentages are weighted and therefore represent the population.

b There were no small urban respondents from DC or RI, no small town respondents from CT, DC, NJ, or RI, and no rural respondents from DC, DE, or NJ.

An appendix table (Table S2) shows the distribution of age, education, overall health, and enrollment status (Medicaid managed care vs. other) by rural/urban categories, both overall and for each racial/ethnic group. Overall, beneficiaries in urban areas were younger, more highly educated, in better health, and more likely to be enrolled in Medicaid managed care plans than beneficiaries in small towns and rural areas (*P*'s < 0.001). Within racial/ethnic groups, there were three notable exceptions to these overall associations. Among AIAN beneficiaries, small town and rural residents were younger than urban residents (*P *=* *0.03). Among API beneficiaries, residents of large urban areas were older than residents of other areas (*P *=* *0.002), and enrollment status (*P *=* *0.51) and overall health (*P *=* *0.65) did not differ by rural/urban categories.

Table 2 presents abridged results from the regression models comparing each racial/ethnic minority group to white beneficiaries on scores on the four composite measures of experiences with care. Scores for the reference group of non‐Hispanic white beneficiaries were higher for doctor communication (*M *=* *72) than for the other three measures, which had means in the range of 51‐58 out of 100. Controlling for differences in age, education, overall health, and state of residence, AIAN beneficiaries reported worse care than white beneficiaries on all measures except doctor communication (*P*'s < 0.001). The largest differences between these two groups were for health plan information and customer service (eight‐point deficit for AIAN beneficiaries) and getting needed care (six‐point deficit). Asian or Pacific Islander beneficiaries reported worse care than white beneficiaries for all four measures (deficits ranged from 13 to 22 points). Compared with white beneficiaries, black beneficiaries reported similar experiences with getting care quickly and better experiences with getting needed care (three‐point advantage, *P *<* *0.001), doctor communication (three‐point advantage, *P *<* *0.001), and health plan customer service (five‐point advantage, *P *<* *0.001). Hispanic beneficiaries reported worse experiences than white beneficiaries on getting needed care and getting care quickly (deficits of 2‐4 points, *P’*s < 0.001), similar experiences with doctor communication, and better experiences with health plan customer service (three‐point advantage, *P *<* *0.001).

**Table 2:** Weighted linear regression models assessing differences in Medicaid beneficiaries’ experiences of care by race/ethnicity

Racial/ethnic group^a^	Getting needed care	Getting care quickly	How well doctors communicate	Health plan information and customer service
	N* *=* *264 060	N* *=* *215 024	N* *=* *187 113	N* *=* *158 291
	*b* (SE)	*b* (SE)	*b* (SE)	*b* (SE)
AIAN	−6.27 (1.43)***	−6.11 (1.64)***	−2.31 (1.79)	−7.94 (1.62)***
API	−19.42 (0.93)***	−22.26 (1.08)***	−13.47 (1.08)***	−18.12 (1.10)***
Black	2.67 (0.28)***	0.76 (0.53)	3.00 (0.45)***	4.87 (0.56)***
Hispanic	−2.49 (0.60)***	−4.10 (0.68)***	1.03 (0.60)	3.49 (0.70)***

	Adjusted mean score (0‐100)	Adjusted mean score (0‐100)	Adjusted mean score (0‐100)	Adjusted mean score (0‐100)
White (reference group)	51.92	58.11	71.85	54.62

Notes:

A total of 2302 respondents were missing information on race/ethnicity and thus were excluded from this analysis. Estimates are from weighted linear regression models predicting each top‐box‐scored measure from race/ethnicity, age, education, overall health, and state of residence. Multiracial beneficiaries were included in the analysis but coefficients comparing this group to white beneficiaries are not shown.

AIAN, American Indian or Alaska Native; API, Asian or Pacific Islander.

*0.01 ≤ *P *<* *0.05; **0.001 ≤ *P *<* *0.01; ***P <* *0.001.

a Each group compared with white.

Table 3 presents abridged results from the regression models comparing API and Hispanic subgroups to white beneficiaries on their experiences with care. Deficits for Asian beneficiaries (who comprise 86% of the API group) on the four dependent measures were slightly larger than the deficits observed in the API group overall. Native Hawaiian or other Pacific Islander white differences were significantly smaller (i.e, less negative) than Asian white differences (*P*'s < 0.001), and the difference between NHOPI and white beneficiaries was only statistically significant for one measure: getting care quickly (10‐point deficit for NHOPI beneficiaries; *P *<* *0.05). Although all Hispanic subgroups were at an advantage relative to white beneficiaries on health plan customer service, the magnitude of the advantage differed significantly by subgroup (*P *<* *0.05); the largest advantage (10 points) was observed among Cuban beneficiaries (who comprise 4% of Hispanics overall) and the smallest (two points) among “other” Hispanics (who comprise 34% of Hispanics overall). Regarding doctor communication, Cuban beneficiaries were at a large advantage (11 points) relative to white beneficiaries (*P *<* *0.001), Mexican beneficiaries (who comprise 46% of Hispanics overall) were at a small advantage (two points, *P *<* *0.05), and Puerto Rican and other Hispanic beneficiaries reported experiences that were similar to those reported by white beneficiaries. On the measures of access to care, Mexican and “other” Hispanic beneficiaries reported worse experiences than white beneficiaries (deficits ranged from 3 to 6 points, *P*'s < 0.001), Puerto Rican beneficiaries (who comprise 16% of Hispanics overall) reported experiences that were similar to those reported by white beneficiaries, and Cuban beneficiaries reported either similar (getting care quickly) or better experiences (getting needed care; six‐point advantage, *P *<* *0.001).

**Table 3:** Weighted linear regression models assessing differences in Medicaid beneficiaries’ experiences of care by racial/ethnic subgroups

Racial/ethnic subgroup^a^	N (%)^b^	Getting needed care	Getting care quickly	How well doctors communicate	Health plan information and customer service
		*b* (SE)	*b* (SE)	*b* (SE)	*b* (SE)
API^c^		***	***	***	***
Asian	10 991 (86.2)	−20.33 (0.95)***	−23.09 (1.10)***	−14.13 (1.10)***	−18.95 (1.13)***
NHOPI	1753 (13.8)	−4.40 (3.44)	−9.56 (4.31)*	−0.65 (3.51)	−3.85 (3.58)
Hispanic^d^		***	***	***	***
Mexican	16 121 (45.6)	−3.00 (0.88)***	−4.43 (1.04)***	2.05 (0.90)*	2.99 (1.03)**
Puerto Rican	5801 (16.4)	−0.72 (1.08)	−1.10 (1.16)	1.38 (0.99)	5.61 (1.15)***
Cuban	1503 (4.3)	6.40 (1.93)***	1.47 (2.08)	10.87 (1.54)***	10.01 (1.90)***
Other Hispanic	11 902 (33.7)	−3.91 (0.91)***	−6.12 (1.03)***	−1.61 (0.90)	2.06 (1.03)*

		Adjusted mean score (0−100)	Adjusted mean score (0−100)	Adjusted mean score (0−100)	Adjusted mean score (0−100)
White (reference group)		51.95	58.15	71.85	54.66

Notes:

A total of 2302 respondents were missing information on race/ethnicity and thus were excluded from this analysis. Estimates are from weighted linear regression models predicting each top‐box‐scored measure from race/ethnicity, age, education, overall health, and state of residence. Black, AIAN, and multiracial beneficiaries were included in the analysis but coefficients comparing these groups to white beneficiaries are not shown.

AIAN, American Indian or Alaska Native; API, Asian or Pacific Islander; NHOPI, Native Hawaiian or other Pacific Islander.

*0.01 ≤ *P *<* *0.05; **0.001 ≤ *P *<* *0.01; ***P <* *0.001.

a Each group compared with white.

b Percent of larger racial/ethnic group of which the subgroup is a part.

c Indicators of statistical significance shown in this row are for a postestimation test of equality of estimates for Asian and NHOPI.

d Indicators of statistical significance shown in this row are for a postestimation joint test of equality of estimates for all Hispanic subgroups.

Table 4 presents abridged results from the regression models comparing beneficiaries in small urban areas, small towns, and rural areas to beneficiaries in large urban areas on the four composite measures of experiences with care. Scores for the reference group of large urban areas were higher for doctor communication (*M *=* *50) than for the other three measures, which had means in the range of 55‐71 out of 100. Controlling for differences in age, education, overall health, and state of residence, beneficiaries in large urban areas reported worse experiences with getting needed care and getting care quickly than did beneficiaries in small urban areas, small towns, and rural areas (deficits ranged from 2 to 3 points on the 0‐100 scale; *P*'s < 0.05). Similarly, beneficiaries in large urban areas reported worse experience getting information and customer service from their plans than did beneficiaries in small towns (*P *<* *0.01).

**Table 4:** Weighted linear regression models assessing differences in Medicaid beneficiaries’ experiences of care by categories of rural/urban residence

Rural/urban category^a^	Getting needed care	Getting care quickly	How well doctors communicate	Health plan information and customer service
	N* *=* *266 179	N* *=* *216 752	N* *=* *188 550	N* *=* *159 720
	*b* (SE)	*b* (SE)	*b* (SE)	*b* (SE)
Small urban	2.39 (0.56)***	1.78 (0.62)**	0.53 (0.53)	1.34 (0.71)
Small town	3.20 (0.68)***	1.80 (0.75)*	1.06 (0.66)	2.46 (0.81)**
Rural	2.33 (0.78)**	2.45 (0.87)**	0.90 (0.75)	0.55 (0.96)

	Adjusted mean score (0‐100)	Adjusted mean score (0‐100)	Adjusted mean score (0‐100)	Adjusted mean score (0‐100)
Large urban (reference group)	49.50	54.77	71.31	54.57

Notes:

A total of 137 respondents were missing information on rural/urban status and thus were excluded from this analysis. Estimates are from weighted linear regression models predicting each top‐box‐scored measure from rural/urban category, age, education, overall health, and state of residence. Geographic groups are based on Rural‐Urban Commuting Area codes: large urban (codes 1‐3), small urban (codes 4‐6), small towns (codes 7‐9), and rural (code 10).

*0.01 ≤ *P *<* *0.05; **0.001 ≤ *P *<* *0.01; ***P <* *0.001.

a Each group compared with large urban areas.

Table 5 presents estimates of differences in care experienced by white beneficiaries vs each of the four superordinate racial/ethnic minority groups based on regression models stratified by rural/urban category. We used postestimation tests to assess whether differences between white beneficiaries and racial/ethnic minority groups varied significantly across rural/urban categories. These tests revealed that racial/ethnic differences were generally similar across rural/urban categories (see Table S3 for details). The only exception was for AIAN‐white differences in getting needed care, which varied significantly by rural/urban category (*P *=* *0.002). Specifically, the disadvantage of AIAN beneficiaries relative to white beneficiaries was only observed in small urban and rural areas (where deficits on getting need care were 9 and 11 points, respectively).

**Table 5:** Weighted racial/ethnic differences in experiences of care by categories of rural/urban residence

	Getting needed care *b* (*SE*)	Getting care quickly *b* (*SE*)	How well doctors communicate *b* (*SE*)	Health plan information and customer service *b* (*SE*)
Large urban				
AIAN vs white	−3.09 (2.11)	−4.05 (2.34)	−2.92 (2.65)	−5.52 (2.39)*
API vs white	−19.00 (0.97)***	−22.51 (1.13)***	−13.49 (1.11)***	−17.77 (1.15)***
Black vs white	3.02 (0.55)***	0.56 (0.60)	3.16 (0.51)***	5.09 (0.63)***
Hispanic vs white	−2.14 (0.66)**	−4.24 (0.75)***	1.31 (0.65)*	3.74 (0.76)***
Small urban				
AIAN vs white	−9.05 (3.16)**	−8.81 (3.95)*	1.21 (3.66)	−11.15 (3.16)***
API vs white	−16.87 (4.58)***	−9.04 (5.47)	−7.97 (3.85)*	−24.44 (3.88)***
Black vs white	5.33 (1.32)***	2.69 (1.46)	2.75 (1.12)*	6.28 (0.49)***
Hispanic vs white	−2.68 (2.07)	−2.81 (2.41)	−1.22 (2.06)	2.99 (1.89)
Small town				
AIAN vs white	−6.38 (4.63)	−0.73 (5.05)	−0.40 (3.32)	−7.30 (2.30)**
API vs white	−18.49 (6.94)**	−2.09 (14.86)	−19.23 (11.73)	−19.58 (0.83)***
Black vs white	3.54 (1.76)*	2.39 (2.13)	4.12 (1.34)**	5.10 (1.53)
Hispanic vs white	−3.55 (3.11)	−2.73 (3.33)	3.49 (2.94)	3.81 (2.34)
Rural				
AIAN vs white	−10.79 (2.27)***	−8.22 (2.85)***	−6.77 (2.80)*	−10.84 (2.50)***
API vs white	8.32 (6.93)	−15.04 (9.86)	2.25 (6.46)	−20.56 (8.20)
Black vs white	1.77 (1.84)	1.55 (1.96)	4.33 (0.97)***	8.17 (1.07)***
Hispanic vs white	2.69 (2.56)	0.88 (2.70)	−1.59 (2.28)	1.00 (2.82)

Notes:

A total of 2436 respondents were missing information on race/ethnicity or rural/urban status and thus were excluded from this analysis. Estimates are from weighted linear regression models predicting each outcome from race/ethnicity (white reference) and all control variables, stratified by rural/urban categories.

AIAN, American Indian or Alaska Native; API, Asian or Pacific Islander.

*0.01 < *P *<* *0.05; **0.001 < *P *<* *0.01; ***P <* *0.001.

## 4 DISCUSSION

Our study contributes substantially to the literature by taking a high‐resolution look at racial/ethnic differences among beneficiaries (including national‐origin subgroups), providing the first examination of urban/rural differences in Medicaid beneficiaries’ experiences with care, and examining how these two factors intersect in predicting experiences with care. We found considerable racial/ethnic differences in Medicaid beneficiaries’ care experiences that broadly mirror differences seen in other populations.^36^, ^37^, ^38^ That is, compared with white beneficiaries, AIAN, API, and Hispanic beneficiaries tended to report worse experiences with access to care, provider communication, and customer service received from plans, though there were some exceptions (e.g, Hispanic beneficiaries reported better experiences with customer service than white beneficiaries).

In contrast, black beneficiaries tended to report better experiences than white beneficiaries. This, too, is consistent with findings from prior studies.^16^, ^37^, ^43^ For example, Weech‐Maldonado and colleagues^43^ investigated patients’ experiences with Medicaid managed care plans in 1999‐2000 and found that, within plans, black beneficiaries reported better experiences with getting needed care, provider communication, and customer services than did white beneficiaries. This raises the question of why other racial/ethnic minorities experience disparities in care when black beneficiaries do not. By restricting our analysis to Medicaid beneficiaries, we have largely accounted for differences in socioeconomic status among racial/ethnic groups that are known to be associated with experiences with care;^44^, ^45^, ^46^ we have not, however, accounted for differences in language barriers. There is reason to believe that language barriers are an even bigger impediment to patient experience for lower‐income minority patients than for higher‐income patients.^16^, ^43^ Thus, black beneficiaries may not show the same deficit in care compared to white beneficiaries in part because they do not face the same language difficulties that may be especially acute in this low‐income population. Though not a new finding, the reason why black beneficiaries score higher than white beneficiaries on some CAHPS measures is not known.

Our analysis of API and Hispanic subgroups uncovered important variation in experiences of care within these larger groups. Notably, NHOPI beneficiaries’ scores on all measures were much higher than Asian beneficiaries’ scores. Future vignette research that assesses whether this group exhibits the same tendency to avoid extreme responses on CAHPS items would help the interpretation of this finding and inform efforts to address disparities. Among Hispanic subgroups, Mexican and “other” Hispanic beneficiaries reported experiences that were similar to Hispanics overall, Puerto Rican beneficiaries’ average experiences rarely differed from those of white beneficiaries, and Cuban beneficiaries reported better experiences than white beneficiaries on three of four measures. Although API and Hispanic subgroups have different geographic distributions and mean socioeconomic status within these larger categories,^47^, ^48^ our analyses controlled for state of residence and thus also state Medicaid programs. Given that, remaining geographic differences probably play a relatively small role in the observed differences by subgroup. Moreover, our analysis was both limited to Medicaid eligible adults and controlled for educational attainment; thus, the effects of additional socioeconomic differences may be small. Additional research is needed to investigate other potential explanations for the subgroup differences reported here.

Medicaid beneficiaries in large urban areas reported poorer experiences with health care access and customer service than did beneficiaries in other areas. This finding suggests that there may be a high concentration of lower‐performing health care providers (where urban Medicaid beneficiaries often seek care) in the urban neighborhoods in which Medicaid beneficiaries tend to be clustered.^49^, ^50^ Another possibility is that urban‐dwelling Medicaid beneficiaries are more likely than other Medicaid beneficiaries to be enrolled in Medicaid managed care plans, which can use utilization management techniques, such as preauthorization policies, that could affect how individuals access care.^51^, ^52^, ^53^ However, the results of a sensitivity test in which we controlled for managed care enrollment in our regression models lent little credence to this hypothesis.

By and large, we found that racial/ethnic and geographic effects were additive rather than multiplicative, suggestion that improvement efforts targeting low‐scoring racial/ethnic groups and geographic regions can proceed independently. The one exception is for AIAN beneficiaries, for whom there appears to be a synergistically negative effect of living in rural areas on getting needed care. Difficulties with getting needed care among rural AIAN beneficiaries may reflect known problems with care delivered by the Indian Health Services (IHS), which is the dominant health care provider to rural AIAN communities.^54^ The rural AIAN population lives almost exclusively in and around reservation lands. In these areas, which are the focus of IHS clinics, lack of transportation affects many patients’ ability to access services. Additionally, 92% of counties with an AIAN majority population, all of which are rural, have been identified as health professional shortage areas, compared with 65% of all rural counties nationally.^33^ Access to nonemergent specialty care services is notably poor in these areas.^55^ When specialty care is required, the IHS often refers patients to larger, contracted hospitals in urban areas, far from the patient's home and community.^56^ Despite their need for specialty care, many of these referred patients cannot find a way to reach that care^56^ Thus, it is perhaps unsurprising that we found AIAN‐white disparities in getting needed care to be especially pronounced in rural areas.

The NAM CAHPS survey was designed to evaluate the health care experiences of a broad set of Medicaid beneficiaries, irrespective of the CMS programs through which those beneficiaries received care. For this reason, we included in our analysis all Medicaid beneficiary types around which the survey was designed, including those who received benefits through both Medicare and Medicaid, that is, dual eligibles and adults with disabilities. We conducted a sensitivity test in which we re‐estimated our regression models without these groups and found that the pattern of findings held even with these two groups omitted. This suggests that the observed racial/ethnic and geographic differences in patient experience are not driven by the Medicare‐based portion of care that some beneficiaries received and are instead broadly applicable to all Medicaid beneficiaries.

Important limitations should be considered when interpreting these results. First, the NAM CAHPS survey was a survey of the community‐dwelling Medicaid population, excluding partial duals and those who qualified for Medicaid via a family planning waiver, and was necessarily restricted to people with known coverage and contact information. It is unknown whether the experiences of other Medicaid beneficiaries would differ, and whether their experiences would have similar or different patterns by race/ethnicity and urbanicity. Second, the NAM CAHPS survey was conducted before the full impact of the ACA could be observed. Follow‐up studies would be helpful for describing how the experiences of Medicaid beneficiaries have changed since 2014‐2015. Third, the set of case mix adjustors used in this study was somewhat restricted. It may be that unobserved differences between racial/ethnic groups or between Medicaid beneficiaries living in different locations account for some of the differences in care experiences observed among these groups. Moreover, although we have characterized Medicaid beneficiaries as being primarily a low‐income population, there is variation in socioeconomic status among this population that has likely not been fully accounted for by our controlling for educational attainment. Fourth, because not all information on race/ethnicity was collected via unassisted self‐report, the gold standard,^57^ some caution is needed in the interpretation of our findings on racial/ethnic differences, as it is possible that results might have differed under conditions of completely self‐reported race/ethnicity. Fifth, although the response rate for the NAM CAHPS survey was typical for surveys of the Medicaid population (including dual eligibles^58^) and research on CAHPS surveys has found little evidence of nonresponse bias after case mix adjustment,^58^, ^59^ it is possible that nonresponse bias influenced our findings. Sixth, this study provides limited insight into the causes of the racial/ethnic and rural/urban differences that were uncovered. Additional research is needed to understand the degree to which these differences reflect geographic isolation, unequal treatment by providers based on patient race or ethnicity, or an increased tendency on the part of certain groups to receive care from providers who are poorer performing overall.^60^ It is possible, in principle, that expectations based on health care received prior to Medicaid might influence one's reference point for an “acceptable” health care experience and result in differences in evaluations associated with race, ethnicity, or geography. In practice, it has been difficult to find evidence that health care expectations differ by race or ethnicity. For example, Weinick and colleagues^61^ found that black, Hispanic, and white respondents reported generally similar health care expectations and provided similar mean responses to CAHPS composites in response to standardized encounters. We are not aware of investigations of possible differences in health care expectations by geography, which may be an important topic for future research.

These limitations notwithstanding, our study suggests improving care for racial/ethnic minorities and those living in urban areas as top priorities for policy makers seeking to improve the quality of care delivered to Medicaid beneficiaries. Our study also suggests a need for improved access to care (i.e, the ability to get care that is needed and to get it in a timely way) for AIAN beneficiaries in small urban and rural areas. Which policy options are likely to be most effective at meeting these needs depends on the reasons that underlie the observed disparities. For example, if transportation barriers among impoverished urban populations drive the geographic differences that we observed, it may be that better urban transportation planning is needed^49^ or that providers need to be further incentivized to live and work in impoverished urban neighborhoods. If, on the other hand, rural/urban differences in care are due to a Medicaid payment system that creates incentives for Medicaid managed care plans to undertreat patients, then raising capitations rates may help to eliminate some of the geographic differences that we observed.^62^ Other strategies are likely to be needed to address racial/ethnic differences in care, including cultural competency training and the provision of language‐appropriate services.

## ENDNOTE

1 Categories for the control variables listed here are the ones used for case mix adjustment. For simplicity, coarser categories were used in describing the sample.
