# Supplementary material for: National racial/ethnic and geographic disparities in experiences with health care among adult Medicaid beneficiaries
Source: Health Serv Res. 2019 Jan 9;54(Suppl 1):287–96. doi: 10.1111/1475-6773.13106 (PMC6341217; doi:10.1111/1475-6773.13106)
Supplement: Supplementary file 2 [file HESR-54-287-s002.docx]

Appendix Tables

Table S1: Survey Items Comprising the Nationwide Adult Medicaid CAHPS Composite Measures Analyzed in This Study

| *Getting Needed Care* |
| --- |
| - In the last six months, how often did you get an appointment to see a specialist as soon as you needed? |
| - In the last six months, how often was it easy to get the care, tests, or treatment you needed? |
| *Getting Care Quickly* |
| - In the last six months, when you needed care right away, how often did you get care as soon as you needed it? |
| - In the last six months, how often did you get an appointment for a check-up or routine care at a doctor’s office or clinic as soon as you needed? |
| *How Well Doctors Communicate* |
| - In the last six months, how often did your personal doctor explain things in a way that was easy to understand? |
| - In the last six months, how often did your personal doctor listen carefully to you? |
| - In the last six months, how often did your personal doctor show respect for what you had to say? |
| - In the last six months, how often did your personal doctor spend enough time with you? |
| *Health Plan Information and Customer Service* |
| - In the last six months, how often did your health plan’s customer service give you the information or help you needed? |
| - In the last six months, how often did your health plan’s customer service staff treat you with courtesy and respect? |

| Table S2: Distribution of Control Variables by Rural/Urban Category, Overall and for Each Racial/Ethnic Group* | | | | | | | | | | | |
| --- | --- | --- | --- | --- | --- | --- | --- | --- | --- | --- | --- |
|  | Overall | | | |  |  | AIAN | | | |  |
|  | Large urban | Small urban | Small town | Rural | *P* value† |  | Large urban | Small urban | Small town | Rural | *P* value† |
| Age, years (%) |  |  |  |  | <0.001 |  |  |  |  |  | 0.034 |
| 18-44 | 52.4 | 53.8 | 51.4 | 48.7 |  |  | 52.5 | 51.3 | 55.1 | 58.1 |  |
| 45-54 | 14.9 | 15.1 | 14.8 | 16.6 |  |  | 16.7 | 14.9 | 17.0 | 17.3 |  |
| 55-64 | 14.4 | 15.9 | 16.1 | 16.7 |  |  | 19.3 | 15.5 | 16.6 | 12.2 |  |
| 65-74 | 9.2 | 8.5 | 9.4 | 10.0 |  |  | 7.9 | 11.2 | 6.9 | 7.2 |  |
| 75 or older | 9.1 | 6.7 | 8.3 | 8.0 |  |  | 3.6 | 7.0 | 4.4 | 5.3 |  |
| Education (%) |  |  |  |  | <0.001 |  |  |  |  |  | 0.030 |
| Less than high school | 32.0 | 30.0 | 31.9 | 30.5 |  |  | 32.2 | 39.9 | 30.9 | 37.1 |  |
| HS degree or GED | 33.0 | 40.1 | 41.9 | 41.4 |  |  | 35.5 | 36.7 | 42.2 | 35.7 |  |
| Some college or 2-yr degree | 25.6 | 25.2 | 21.6 | 23.5 |  |  | 28.2 | 20.7 | 25.4 | 24.8 |  |
| 4-yr college degree or more | 9.3 | 4.7 | 4.6 | 4.6 |  |  | 4.0 | 2.7 | 1.5 | 2.5 |  |
| Overall health (%) |  |  |  |  | <0.001 |  |  |  |  |  | 0.792 |
| Excellent | 10.0 | 7.8 | 7.1 | 7.2 |  |  | 9.2 | 8.5 | 8.5 | 11.7 |  |
| Very good | 20.4 | 18.2 | 17.2 | 19.1 |  |  | 18.6 | 15.3 | 20.5 | 17.5 |  |
| Good | 33.4 | 32.3 | 31.0 | 31.1 |  |  | 33.1 | 32.8 | 30.3 | 34.4 |  |
| Fair | 27.1 | 30.0 | 31.7 | 30.0 |  |  | 27.7 | 30.5 | 29.8 | 28.2 |  |
| Poor | 9.1 | 11.8 | 12.9 | 12.5 |  |  | 11.3 | 12.8 | 11.0 | 8.2 |  |
| Enrollment status (%) |  |  |  |  | <0.001 |  |  |  |  |  | <0.001 |
| Managed care | 36.70 | 29.34 | 23.49 | 22.12 |  |  | 24.70 | 9.75 | 11.91 | 6.95 |  |
| Other | 63.30 | 70.66 | 76.51 | 77.88 |  |  | 75.30 | 90.25 | 88.09 | 93.05 |  |
| *Notes*: 2,436 respondents were missing information on race/ethnicity and/or rural/urban status and thus were excluded from this analysis. Multiracial beneficiaries’ results are not shown separately but are included in the overall results. Geographic groups are based on Rural-Urban Commuting Area codes: Large urban (codes 1-3), Small urban (codes 4-6), Small towns (codes 7-9), and Rural (code 10). Results are weighted.  AIAN = American Indian or Alaska Native; API = Asian or Pacific Islander  * Percentages are weighted and therefore represent the population.  † Rao-Scott chi-square test were run for each characteristic by rural/urban category within race/ethnicity. | | | | | | | | | | | |

| Table S2 (continued): Distribution of Control Variables by Rural/Urban Category, Overall and for Each Racial/Ethnic Group* | | | | | | | | | | | |
| --- | --- | --- | --- | --- | --- | --- | --- | --- | --- | --- | --- |
|  | API | | | |  |  | Black | | | |  |
|  | Large urban | Small urban | Small town | Rural | *P* value† |  | Large urban | Small urban | Small town | Rural | *P* value† |
| Age, years (%) |  |  |  |  | 0.002 |  |  |  |  |  | <0.001 |
| 18-44 | 42.3 | 53.0 | 63.1 | 44.1 |  |  | 50.6 | 48.6 | 48.2 | 44.6 |  |
| 45-54 | 11.8 | 10.5 | 12.8 | 7.5 |  |  | 16.3 | 14.8 | 13.5 | 15.7 |  |
| 55-64 | 9.7 | 11.6 | 7.7 | 19.5 |  |  | 17.8 | 18.8 | 18.5 | 15.9 |  |
| 65-74 | 14.5 | 8.3 | 6.2 | 9.3 |  |  | 8.5 | 9.5 | 9.8 | 11.3 |  |
| 75 or older | 21.6 | 16.7 | 10.2 | 19.6 |  |  | 6.7 | 8.3 | 10.0 | 12.4 |  |
| Education (%) |  |  |  |  | <0.001 |  |  |  |  |  | <0.001 |
| Less than high school | 35.2 | 27.0 | 24.3 | 29.4 |  |  | 29.3 | 35.9 | 38.7 | 43.1 |  |
| HS degree or GED | 23.4 | 41.8 | 39.3 | 43.8 |  |  | 37.3 | 37.9 | 41.5 | 36.8 |  |
| Some college or 2-yr degree | 21.6 | 22.3 | 27.1 | 18.5 |  |  | 26.7 | 23.2 | 17.0 | 17.3 |  |
| 4-yr college degree or more | 19.8 | 9.0 | 9.4 | 8.2 |  |  | 6.7 | 3.0 | 2.7 | 2.8 |  |
| Overall health (%) |  |  |  |  | 0.651 |  |  |  |  |  | <0.001 |
| Excellent | 9.4 | 12.2 | 6.8 | 11.7 |  |  | 10.6 | 8.9 | 7.5 | 8.2 |  |
| Very good | 23.0 | 21.3 | 37.2 | 16.6 |  |  | 20.5 | 14.9 | 14.3 | 18.4 |  |
| Good | 41.0 | 38.7 | 35.0 | 43.7 |  |  | 31.5 | 31.0 | 31.6 | 30.6 |  |
| Fair | 21.5 | 23.9 | 18.7 | 24.1 |  |  | 29.7 | 35.4 | 36.2 | 33.3 |  |
| Poor | 5.2 | 3.9 | 2.2 | 4.0 |  |  | 7.8 | 9.9 | 10.4 | 9.4 |  |
| Enrollment status (%) |  |  |  |  | 0.514 |  |  |  |  |  | <0.001 |
| Managed care | 48.43 | 50.91 | 58.60 | 55.45 |  |  | 34.78 | 18.31 | 16.85 | 15.87 |  |
| Other | 51.57 | 49.09 | 41.40 | 44.55 |  |  | 65.22 | 81.69 | 83.15 | 84.13 |  |
| *Notes*. 2,436 respondents were missing information on race/ethnicity and/or rural/urban status and thus were excluded from this analysis. Multiracial beneficiaries’ results are not shown separately but are included in the overall results. Geographic groups are based on Rural-Urban Commuting Area codes: Large urban (codes 1-3), Small urban (codes 4-6), Small towns (codes 7-9), and Rural (code 10). Results are weighted.  AIAN = American Indian or Alaska Native; API = Asian or Pacific Islander  * Percentages are weighted and therefore represent the population.  † Rao-Scott chi-square test were run for each characteristic by rural/urban category within race/ethnicity. | | | | | | | | | | | |

| Table S2 (continued): Distribution of Control Variables by Rural/Urban Category, Overall and for Each Racial/Ethnic Group* | | | | | | | | | | | |
| --- | --- | --- | --- | --- | --- | --- | --- | --- | --- | --- | --- |
|  | Hispanic | | | |  |  | White | | | |  |
|  | Large urban | Small urban | Small town | Rural | *P* value† |  | Large urban | Small urban | Small town | Rural | *P* value† |
| Age, years (%) |  |  |  |  | 0.028 |  |  |  |  |  | <0.001 |
| 18-44 | 57.8 | 60.1 | 53.3 | 51.9 |  |  | 51.2 | 53.5 | 51.3 | 48.3 |  |
| 45-54 | 13.4 | 11.6 | 12.6 | 16.1 |  |  | 15.9 | 16.0 | 15.2 | 16.3 |  |
| 55-64 | 10.4 | 12.1 | 13.0 | 14.2 |  |  | 16.4 | 16.2 | 16.0 | 17.6 |  |
| 65-74 | 9.2 | 8.7 | 9.1 | 10.3 |  |  | 8.6 | 8.0 | 9.5 | 10.0 |  |
| 75 or older | 9.3 | 7.5 | 12.0 | 7.5 |  |  | 7.9 | 6.2 | 8.0 | 7.9 |  |
| Education (%) |  |  |  |  | <0.001 |  |  |  |  |  | <0.001 |
| Less than high school | 47.1 | 45.0 | 44.9 | 42.3 |  |  | 22.1 | 26.1 | 29.3 | 27.5 |  |
| HS degree or GED | 26.8 | 31.5 | 40.7 | 35.9 |  |  | 38.0 | 42.8 | 42.5 | 43.9 |  |
| Some college or 2-yr degree | 20.1 | 19.2 | 10.6 | 19.1 |  |  | 29.0 | 26.1 | 23.0 | 23.6 |  |
| 4-yr college degree or more | 6.0 | 4.3 | 3.8 | 2.7 |  |  | 10.9 | 5.0 | 5.2 | 5.0 |  |
| Overall health (%) |  |  |  |  | 0.071 |  |  |  |  |  | <0.001 |
| Excellent | 11.1 | 11.1 | 10.4 | 11.8 |  |  | 8.7 | 6.6 | 6.3 | 6.2 |  |
| Very good | 18.3 | 16.6 | 19.7 | 15.4 |  |  | 21.3 | 19.1 | 17.5 | 19.9 |  |
| Good | 34.5 | 36.3 | 24.8 | 31.8 |  |  | 32.4 | 32.2 | 31.9 | 31.3 |  |
| Fair | 27.9 | 27.9 | 35.8 | 30.3 |  |  | 26.6 | 29.4 | 30.6 | 29.6 |  |
| Poor | 8.1 | 8.0 | 9.2 | 10.6 |  |  | 11.0 | 12.7 | 13.7 | 12.9 |  |
| Enrollment status (%) |  |  |  |  | <0.001 |  |  |  |  |  | <0.001 |
| Managed care | 41.08 | 37.89 | 32.12 | 29.64 |  |  | 30.90 | 29.59 | 23.88 | 22.58 |  |
| Other | 58.92 | 62.11 | 67.88 | 70.36 |  |  | 69.10 | 70.41 | 76.12 | 77.42 |  |
| *Notes*: 2,436 respondents were missing information on race/ethnicity and/or rural/urban status and thus were excluded from this analysis. Multiracial beneficiaries’ results are not shown separately but are included in the overall results. Geographic groups are based on Rural-Urban Commuting Area codes: Large urban (codes 1-3), Small urban (codes 4-6), Small towns (codes 7-9), and Rural (code 10). Results are weighted.  AIAN = American Indian or Alaska Native; API = Asian or Pacific Islander  * Percentages are weighted and therefore represent the population.  † Rao-Scott chi-square test were run for each characteristic by rural/urban category within race/ethnicity. | | | | | | | | | | | |

Table S3: Weighted Results of Post-Estimation Tests of Whether Racial/Ethnic Differences in Experiences with Care Vary by Rural/Urban Category

|  | Getting needed care | Getting care quickly | How well doctors communicate | Health plan information and customer service |
| --- | --- | --- | --- | --- |
|  | *p* | *p* | *p* | *p* |
| AIAN-White difference | 0.002 | 0.063 | 0.737 | 0.109 |
| API-White difference | 0.139 | 0.088 | 0.075 | 0.544 |
| Black-White difference | 0.473 | 0.158 | 0.833 | 0.579 |
| Hispanic-White difference | 0.594 | 0.557 | 0.322 | 0.419 |
| *Notes*: 2,436 respondents were missing information on race/ethnicity and/or rural/urban status and thus were excluded from this analysis. Multiracial beneficiaries were included in the analysis but coefficients comparing this group to Whites are not shown. Based on weighted linear regression models predicting each outcome from race/ethnicity (White reference), rural/urban category (large urban reference), the interaction between race/ethnicity and rural/urban category, and all control variables, we ran post-estimation joint-hypothesis tests to assess whether differences between Whites and racial/ethnic minority groups varied significantly across rural/urban category. This table presents *p* values for each of those joint-hypothesis tests. | | | | |
